# Identification of glycolysis related pathways in pancreatic adenocarcinoma and liver hepatocellular carcinoma based on TCGA and GEO datasets

**DOI:** 10.1186/s12935-021-01809-y

**Published:** 2021-02-19

**Authors:** Ji Li, Chen Zhu, Peipei Yue, Tianyu Zheng, Yan Li, Biao Wang, Xin Meng, Yao Zhang

**Affiliations:** 1 Department of Radiation Oncology, Cancer Hospital of China Medical University, Liaoning Cancer Hospital & Institute, No.44 Xiaoheyan Road, Dadong District, Shenyang, Liaoning China; 2 Department of Neurosurgery, The First Hospital of China Medical University, Shenyang, Liaoning China; 3 Department of Biochemistry and Molecular Biology, College of Life Science, China Medical University, Shenyang, Liaoning China; 4 Department of Laboratory Medicine, The Fourth Affiliated Hospital of China Medical University, Chongshan East Street, Shenyang, Liaoning China; 5 The VIP Department, School and Hospital of Stomatology, Liaoning Provincial Key Laboratory of Oral Diseases, China Medical University, Shenyang, Liaoning China; 6 Department of General Surgery, The Fourth Affiliated Hospital of China Medical University, Chongshan East Street, Shenyang, Liaoning China; 7 Department of gynaecology, Shengjing Hospital of China Medical University, NO.36 Sanhao street, Heping district, 110000 Shenyang, Liaoning China

**Keywords:** Digestive system tumors, Glycolysis, Signaling pathway, Prognosis

## Abstract

**Background:**

Abnormal energy metabolism is one of the characteristics of tumor cells, and it is also a research hotspot in recent years. Due to the complexity of digestive system structure, the frequency of tumor is relatively high. We aim to clarify the prognostic significance of energy metabolism in digestive system tumors and the underlying mechanisms.

**Methods:**

Gene set variance analysis (GSVA) R package was used to establish the metabolic score, and the score was used to represent the metabolic level. The relationship between the metabolism and prognosis of digestive system tumors was explored using the Cancer Genome Atlas (TCGA) and Gene Expression Omnibus (GEO) databases. Volcano plots and gene ontology (GO) analyze were used to show different genes and different functions enriched between different glycolysis levels, and GSEA was used to analyze the pathway enrichment. Nomogram was constructed by R package based on gene characteristics and clinical parameters. qPCR and Western Blot were applied to analyze gene expression. All statistical analyses were conducted using SPSS, GraphPad Prism 7, and R software. All validated experiments were performed three times independently.

**Results:**

High glycolysis metabolism score was significantly associated with poor prognosis in pancreatic adenocarcinoma (PAAD) and liver hepatocellular carcinoma (LIHC). The STAT3 (signal transducer and activator of transcription 3) and YAP1 (Yes1-associated transcriptional regulator) pathways were the most critical signaling pathways in glycolysis modulation in PAAD and LIHC, respectively. Interestingly, elevated glycolysis levels could also enhance STAT3 and YAP1 activity in PAAD and LIHC cells, respectively, forming a positive feedback loop.

**Conclusions:**

Our results may provide new insights into the indispensable role of glycolysis metabolism in digestive system tumors and guide the direction of future metabolism–signaling target combined therapy.

## Introduction

Digestive system tumors are the most common type of tumor, and have become a major human health hazard owing to their high morbidity and mortality [1–4]. Due to the features of local recurrence, lymph node invasion, and distant metastasis, the prognosis of patients with digestive system tumors remains unsatisfactory even after maximum surgical resection combined with radiotherapy and chemotherapy, especially for pancreatic adenocarcinoma (PAAD) and liver hepatocellular carcinoma (LIHC). As we all know, PAAD and LIHC are highly malignant tumors, which are the leading causes of cancer death all over the world, and patients are usually diagnosed at advanced stages. There is an urgent need to establish a stable survival-related predictive system for improving malignancy evaluation and guiding treatment.

Energy metabolism reprogramming can promote cell growth and proliferation, and has been recognized as a new cancer marker [5]. Compared with normal cells, cancer cells environmental conditions have heterogeneous spaces and conditions, and there are great differences in glycolysis, glutamine, and lipid levels. Changes in cancer cells metabolism can make them adapt better to the tumor microenvironment and survive [6]. Tumor is a metabolic disease, tumor cells must constantly increase the synthesis of new substances and maintain the energy supply to sustain proliferation and distant metastasis. Different tumor types may have different metabolisms, moreover, different stages of the same cancer type may also have differing metabolisms. Unlike normal cell metabolism, most cancer cells rely, even under aerobic conditions, mainly on aerobic glycolysis for glycolysis metabolism, which is termed the Warburg effect [7]. Aerobic glycolysis yields ATP and lactic acid quickly to promote tumor proliferation and invasion, which could be considered a potential target for anti-cancer therapies. Metabolic abnormalities often indicate tumor abnormalities. [18F]-fluorodeoxyglucose positron emission tomography-computed tomography (FDG PET/CT) scans can identify malignant lesions by targeting the high glycolysis rate of cancer cells [8]. Therefore, it is expected that metabolism will become an intervention target in digestive system cancer therapy. Especially in PAAD and LIHC, both pancreas and liver are large digestive glands of the body, with complex structure and a variety of digestive fluids secreting. Besides, liver is also the metabolic factory of the body, which may lead to PAAD and LIHC being more sensitive to glycolysis. However, a single factor is often not a good evaluation of the state of the tumor, so there is an urgent need to evaluate metabolic criteria via a series of indicators.

Recent studies have found that several classical pathways can regulate metabolism, and metabolism status can also affect pathway activation [9–12]. Mammalian target of rapamycin–signal transducer and activator of transcription 3 (mTOR–STAT3) can inhibit liver cancer cell glycolysis by inhibiting hexokinase 2 (HK2). We have also found that, in hepatocellular carcinoma cells under hypoxic conditions, Yes1-associated protein (YAP1) and hypoxia-inducible factor 1α (HIF-1α) bind to activate pyruvate kinase M1/2 (PKM2) to promote glycolysis [11]. In nasopharyngeal carcinoma, forkhead box C2 (FOXC2) can activate YAP and regulate HK2 positively to promote glycolysis [13]. Enzo et al. found that, in breast cancer, 2-deoxyglucose (2-DG) could inhibit expression of the YAP/Tafazzin (TAZ)-related genes [10]. However, whether metabolism and pathway can regulate each other is still unclear.

With the advent of big data, the development of bioinformatics has shed light on the biological secrets endowed by huge and complicated biological data. The completion of the Human Genome Project has uncovered the secret of 3 billion base pairs of 25,000 human genes, maximized data sharing, and greatly accelerated biomedical research [14]. According to 16S rRNA gene sequencing, pancreatic adenocarcinoma patients with short or long-term survival have different tumor microbiome composition, and survival time may be predicted by microbiome characteristics [15]. Wungki Park et al. used a genomic approach to identify and define the status of homologous recombination deficiency (HRD) in pancreatic cancer patients and assess their association with DNA damage-targeted therapy, so as to maximize therapeutic outcomes [16]. Multiple data platforms were integrated to conduct comprehensive analysis on gene mutation, methylation, copy number variation, and immunophenotype of hepatocellular carcinoma patients, so as to better understand the molecular targets of treatment and improve treatment strategies [17]. Increasing evidence shows that metabolism-related genes can be as biomarkers for predicting prognosis. In the present study, we used bioinformatics to evaluate the whole metabolism in digestive system cancers, revealing the relationship between the metabolic score and prognosis, as well as the critical driving pathways of malignancy. Our study may provide new guidance and insights for the clinical diagnosis and intervention of digestive system tumors.

## Materials and methods

### Data source

The training set comprised the mRNA expression profiles and the corresponding clinical information from The Cancer Genome Atlas (TCGA) database (http://cancergemome.nih.gov/). The validation set was comprised of two public datasets (GSE57495, GSE14520) downloaded from the Gene Expression Omnibus (GEO) database (http://www.ncbi.nlm.nih.gov/geo/).

### Bioinformatics analysis

#### GSVA score construction

Metabolic gene sets were downloaded from the molecular signature database (MSigDB, http://software.broadinstitute.org/gsea/msigdb). First, we downloaded the most appropriate gene sets of glycolysis, fatty acid and glutamine metabolism respectively. Then, the gene set variance analysis (GSVA) scores of each gene set for each sample in TCGA and GEO databases were obtained using the GSVA R package. The GSVA score could represent the degree of enrichment of these metabolic related genes, that is to say, each patient has a GSVA score, which can reflect the metabolic level. We named these GSVA scores as “glycolytic score”, “fatty acid score” and “glutamine score” respectively. Additional file 1: Table S1 shows the corresponding gene sets.

####  Survival analyze

High- and low-risk groups were defined according to the median GSVA score [18]. Survival curves showed the effects of different metabolic types and metabolic levels on the prognosis value of the four digestive system cancers.

#### Gene ontology (GO) analysis

Patients were divided into high-risk and low-risk groups based on the median glycolysis GSVA score, the differentially expressed genes (DEG) between the two groups were explored using the limma R package. And it was shown as a volcano map. In order to explore which functions or biological processes are related to glycolysis level, we conducted GO analysis. The differentially expressed genes (logFC > 2, P < 0.05) were introduced into the David website, the function annotation was analyzed using gene ontology (GO) (http://david.ncifcrf.gov/) [19].

#### Gene set enrichment analysis (GSEA)

GSEA was performed to identify the enrichment of specific gene sets [20]. In TCGA-PAAD or LIHC datasets, patient samples were divided into two group (high-risk and low-risk group) according to glycolysis GSVA score, according to the all genes expression in each of the two groups, GSEA software 3.0 was utilized to identify underlying molecular mechanisms. We consider the value of |NES| > 1.5, P < 0.05 as the standard of significant enrichment.

#### Construction and assessment of the nomogram

Glycolysis risk score combined with clinicopathologic features by nomogram were used to predict 3- and 5-year survival rates of patients with PAAD and LIHC via “rms” R software package. calibration plot by R package shown the accuracy of predicting survival by nomogram.

### Statistical analysis

The patients were divided into high- and low-risk groups based on the risk score. The difference in overall survival (OS) between the two groups was evaluated by Kaplan-Meier survival analysis and the log-rank test. All statistical analyses were performed using SPSS, GraphPad Prism 7, or R software. P < 0.05 was considered statistically significant.

### Cell culture

BxPC-3 and Huh7 cells were obtained from the Cell Culture Center of the Chinese Academy of Sciences (Shanghai, China). BxPC-3 cells and Huh7 cells were cultured in RPMI 1640 medium and modified Eagle’s medium (Gibco), respectively, supplemented with 100 U/mL penicillin, 1 µg/mL streptomycin (Gibco), and 10 % fetal bovine serum (Procell) at 37 °C with 5 % CO_2_.

### RNA extraction and quantitative PCR (qPCR)

RNA was extracted using TRIzol(Takara) according to the manufacturer’s protocol. The total RNA was reverse-transcribed into complementary DNA (cDNA) using a PrimeScript RT Reagent Kit (Takara). RT-qPCR was performed using ChamQ Universal SYBR qPCR Master Mix (Vazyme) on a LightCycler 480. 18S was used as an internal control, and other expression levels were always given relative to that of 18S.

### Western blotting

Total protein was collected by Radio-immunoprecipitation assay buffer with phosphatase inhibitor, and quantified by a bicinchoninic acid protein assay kit (Solarbio). Then, the protein was separated by 10 % sodium dodecyl sulfate–polyacrylamide gel electrophoresis (Wanleibio), transferred to 0.45-µm polyvinylidene fluoride membranes (Millipore), and blocked with 5 % skim milk (BD) for 2 h at room temperature. The membranes were incubated overnight with the appropriate primary antibodies (YAP1:1/1000, Cell Signaling Technology (CST)#14,074; P-YAP1 (Ser127): 1/1000, CST#13,008; HK2: 1/1000, CST#2867; PKM2: 1/1000, CST#4053; STAT3: 1/1000, Wanleibio#WL01836; P-STAT3(Ser727): 1/1000, Wanleibio#WLP2412; VEGFA:1/1000, Proteintech#66,828; β-Actin: 1/10,000, Proteintech#60008-1-Ig) at 4 °C. The next day, the membranes were washed with Tris-buffered saline containing Tween 20 (TBST), then incubated with the appropriate secondary antibodies (1/10,000, CST) for 1 h at room temperature. Subsequently, the membranes were washed with TBST and the bands were detected using an ECL Western Blot Detection Kit (Wanleibio).

### Pathway inhibitors

Inhibitors of STAT3 (C188-9, MCE#HY-112,288) and YAP1 (verteporfin, MCE#HY-B0146) were dissolved in dimethyl sulfoxide. BxPC-3 cells were treated with 20 µM and 50 µM C188-9 for 6 h, and then cultured for 24 h. Huh7 cells were treated with 1 µM and 2 µM verteporfin for 6 h, and then cultured for another 24 h. siYAP was transfected into Huh7 cells for 24 h. Sequences of siRNA were shown in Additional file 1:  Table S2.

### Intervention of glycolysis

Sodium pyruvate (Sigma, p2256) was used to mimic the glycolysis effect. We weakened the progression of glycolysis by adding 2-DG (Sigma, D8375) (Additional file 2).

###  Glycolysis and lactate level measurement

Glucose uptake and lactate production were detected using a High Sensitivity Glycolysis Assay Kit (Sigma, MAK181) and Lactate Assay Kit (Sigma, MAK065), respectively, according to the manufacturer’s instructions.

## Results

### Prognostic value of metabolism signature in digestive system cancers

**Fig. 1:**
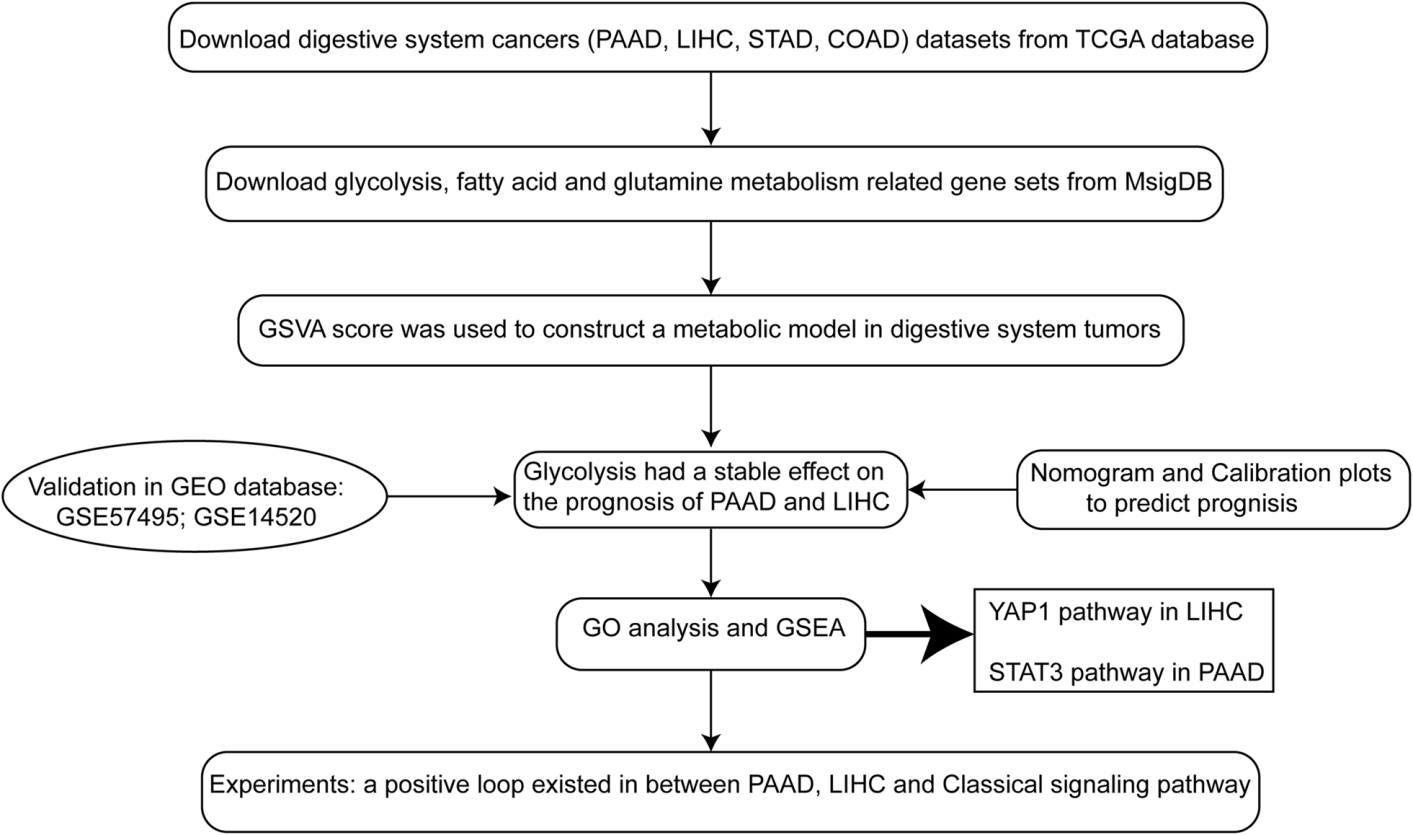
Flow chart of this study

The flow chart of our study was shown in Fig. 1. We downloaded the datasets of digestive system tumors (pancreatic adenocarcinoma, liver hepatocellular carcinoma, stomach adenocarcinoma and colon adenocarcinoma) from TCGA database, and the most appropriate gene sets of glycolysis, fatty acid and glutamine metabolism downloaded from MSigDB. GSVA score was used to calculate the enrichment of glycolysis, fatty acid metabolism and glutamine metabolism genes in each patient in TCGA, it was found that glycolysis has a universally applicable clinical prognostic value in digestive tumors, especially in PAAD and LIHC, it is also verified in the GEO database. And nomogram and calibration plot shown the accuracy of predicting survival in PAAD and LIHC. Moreover, we found that there was a positive feedback loop between glycolysis and the classical signaling pathway, which was verified by a series of experiments.

**Fig. 2:**
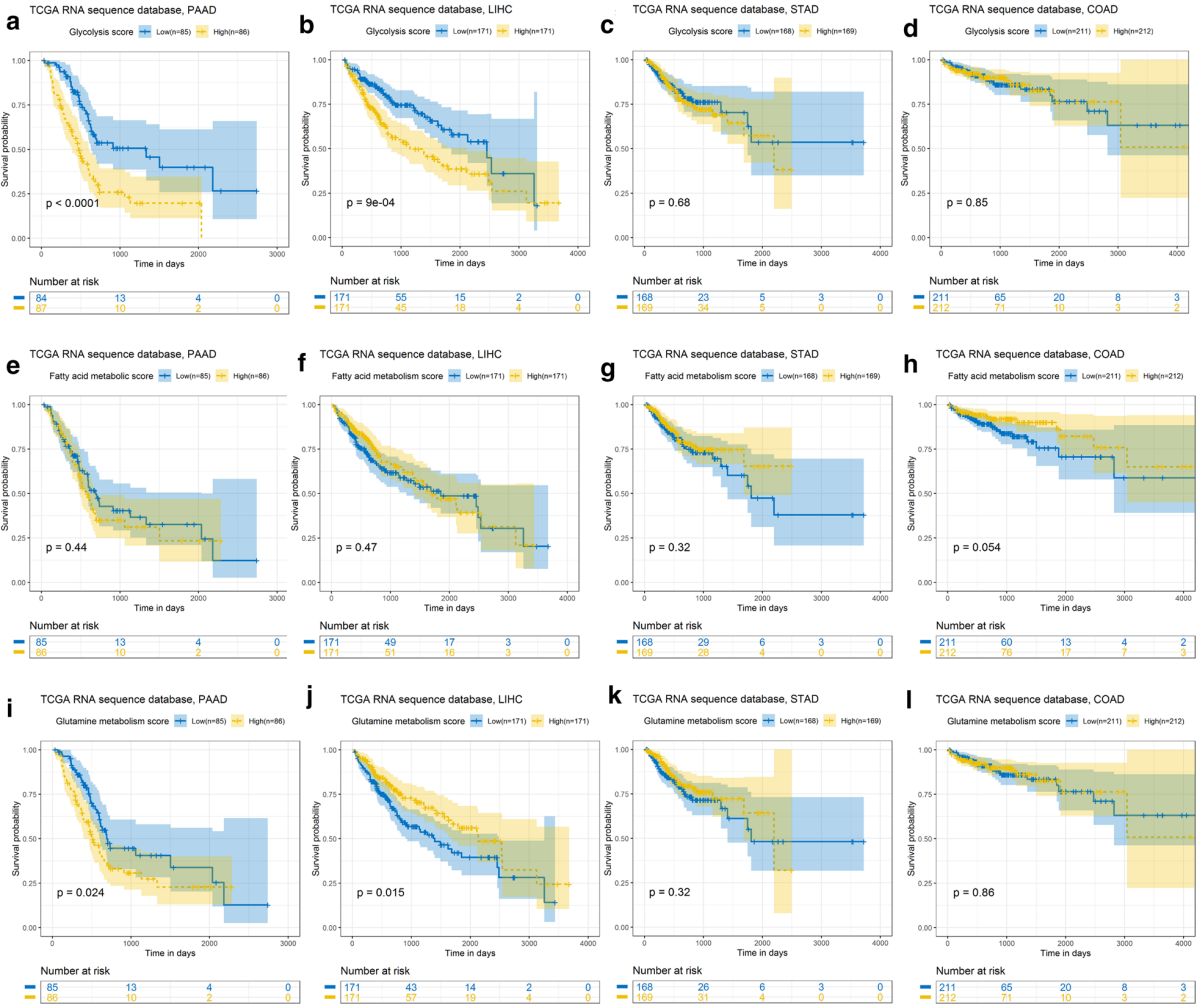
Prognostic value of the metabolic signature in digestive system cancer. **a–d** OS analysis of the glycolysis-related signature in PAAD (**a**) LIHC (**b**) STAD (**c**), and COAD (**d**). **e–h** OS analysis of the lipid metabolism–related signature in PAAD (**e**), LIHC (**f**), STAD (**g**), and COAD (**h**). **i–l** OS analysis of glutamine metabolism–related signature in PAAD **(i)**, LIHC **(j)**, STAD (**k**), and COAD (**l**)

**Fig. 3:**
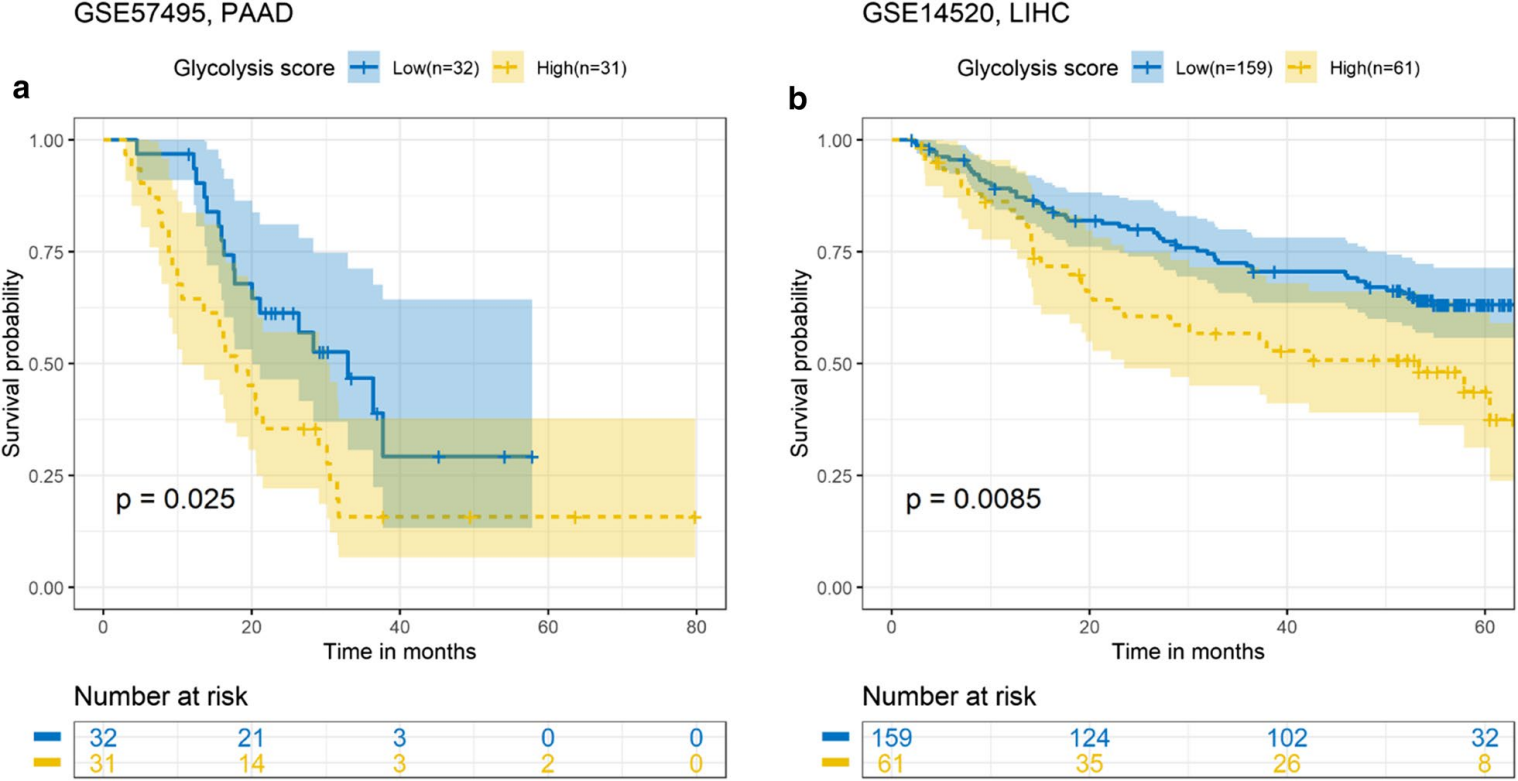
Prognostic value of the glucose metabolic signature in GEO validation sets. High risk scores indicated poor prognosis in PAAD (**a**) and LIHC (**b**)

To explore the relation of metabolic status and digestive system cancers, we downloaded four of the most common digestive system cancers from TCGA database: PAAD, LIHC, stomach adenocarcinoma (STAD), and colon adenocarcinoma (COAD). Analysis of the patients’ metabolic status score and survival showed that the influence of glycolysis metabolism on the four cancers had a relatively stable trend. Especially in PAAD and LIHC, the high-risk group had significantly lower OS time than the low-risk group (P < 0.001) (Fig. 2a–d). Moreover, consistent results were obtained for the PAAD and LIHC GEO validation sets (Fig. 3a, b). Glutamine metabolism had opposite effects on the prognosis of patients with PAAD and LIHC. In PAAD, the high-risk group had longer survival time than the low-risk group; in LIHC, the low-risk group had better prognosis (Fig. 2i–l). However, the fatty acid metabolism score had no significant effect on prognosis (Fig. 2e–h).

### Glycolysis‐related signature is associated with pathological features in PAAD and LIHC

**Fig. 4:**
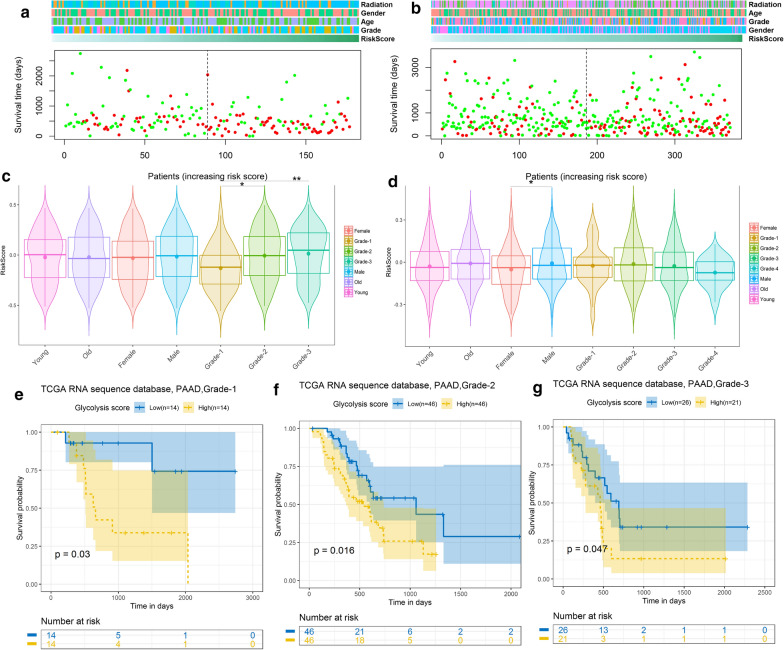
Correlation between glycolysis metabolism and pathological characteristics. (**a, b**) Survival scatter plot (red: dead, green: alive) of glycolysis metabolism risk score correlated with OS in PAAD (**a**) and LIHC (**b**). **c, d** Violin plot of risk score correlated with clinical pathological feature in PAAD (**c**) and LIHC (**d**). **e–g** Prognostic value of glycolysis metabolism in different clinical stages of PAAD. *P < 0.05, **P < 0.01

We choose the glycolysis metabolism for the subsequent analyses because it had a relatively consistent prognosis for the patients in most digestive system cancer. We explored the relation between glycolysis metabolism status and clinical pathological features in PAAD and LIHC (Fig. 4a and b). We also analyzed the glycolysis related genes that were significantly difference in different glycolysis groups and showed the top10 using a histogram (Additional file 1: Fig. S1a, b). Patients were arrayed based on their GSVA glycolysis metabolism risk scores. There was no direct relationship between risk score and patient age and gender. Interestingly, increased PAAD grade was accompanied by gradually increased scores, but this was not found for LIHC (Fig. 4c, d). Subsequently, we compared the risk scores in different grades of PAAD, where the high-risk group was still associated with poor prognosis (Fig. 4e–g).

To evaluate whether the glycolysis metabolism signature can be an independent prognostic factor in patients with digestive system cancer, we performed univariate and multivariate Cox regression analyses for the pancreatic and hepatocellular carcinomas. In pancreatic cancer, glycolysis metabolism and radiotherapy could be considered independent factors related to survival (Table 1); in LIHC, the glycolysis metabolism signature and disease stage could be considered independent factors for evaluating survival (Table 2).

**Table 1:** Cox regression analysis of TCGA, PAAD

Characeristics	Univariate analysis		Multivariate analysis	
	HR	P value	HR	P value
Age				
(young < 65 VS old ≥ 65)	0.7627	0.198		
Gender				
(Male VS Female)	0.82	0.343		
Radiation therapy				
(Yes VS No)	0.3906	0.00614	0.4370	0.0180
Grade				
(High VS Low)	2.181	0.0171	2.0803	0.1288
Stage				
(High VS Low)	2.3079	0.0355	2.2221	0.1386
Risk score				
(High VS Low)	4.8713	0.00029	0.00029	0.0180

*TCGA* The Cancer Genome Atlas,* PAAD* pancreatic adenocarcinoma,* HR* hazard ratio

**Table 2:** Cox regression analysis of TCGA, LIHC

Characeristics	Univariate analysis		Multivariate analysis	
	HR	P value	HR	P value
Age				
(young < 65 VS old ≥ 65)	0.7882	0.18		
Gender				
(Male VS Female)	0.8148	0.257		
Radiation therapy				
(Yes VS No)	1.605	0.641		
Grade				
(High VS Low)	1.12	0.539		
Stage				
(High VS Low)	2.0737	0.000168	1.9181	0.000911
Risk score				
(High VS Low)	6.8069	0.000758	4.4964	0.012725

*TCGA* The Cancer Genome Atlas,*LIHC* Hepatocellular carcinoma* HR* Hazard ratio

### Building a nomogram for predicting survival in PAAD and LIHC patients

**Fig. 5:**
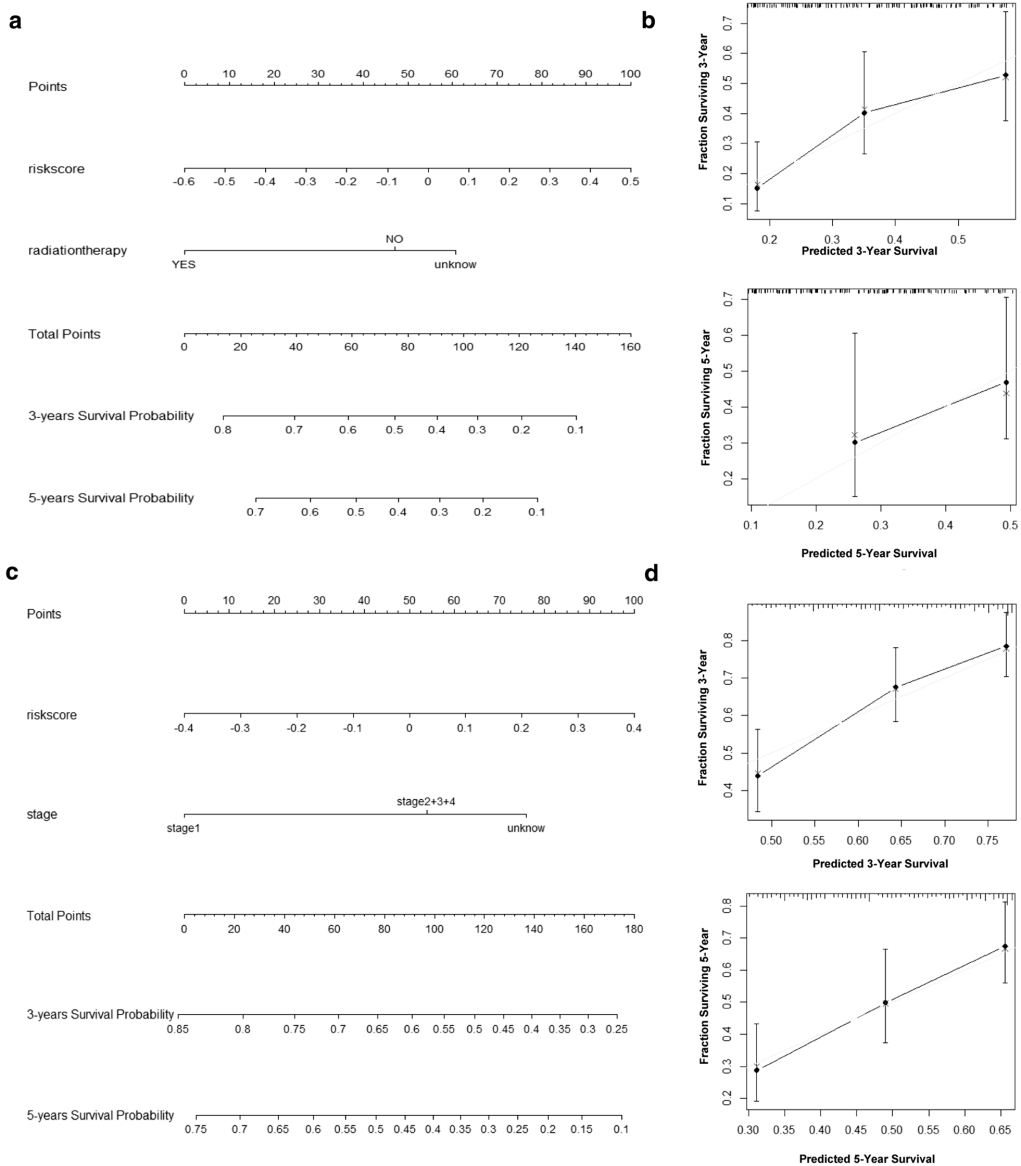
Nomograms predicting overall survival. The nomogram to predict 3- and 5-year OS in PAAD (**a**) and LIHC (**c**). The calibration plot for validation of the nomogram in PAAD (**b**) and LIHC (**d**)

In order to more easily evaluate the prognosis of clinical cancer patients, we used the risk score in combination with other independent prognostic factors to establish nomograms in PAAD (Fig. 5a) and LIHC (Fig. 5c) patients, respectively, nomogram showed the 3-year, 5-year likelihood of survival. The Calibration plots also evaluated the accuracy of the nomogram prediction and found a good agreement with the ideal model (gray curve) (Fig. 5b and d).

### Functional analysis of glycolysis metabolism–associated signatures

**Fig. 6:**
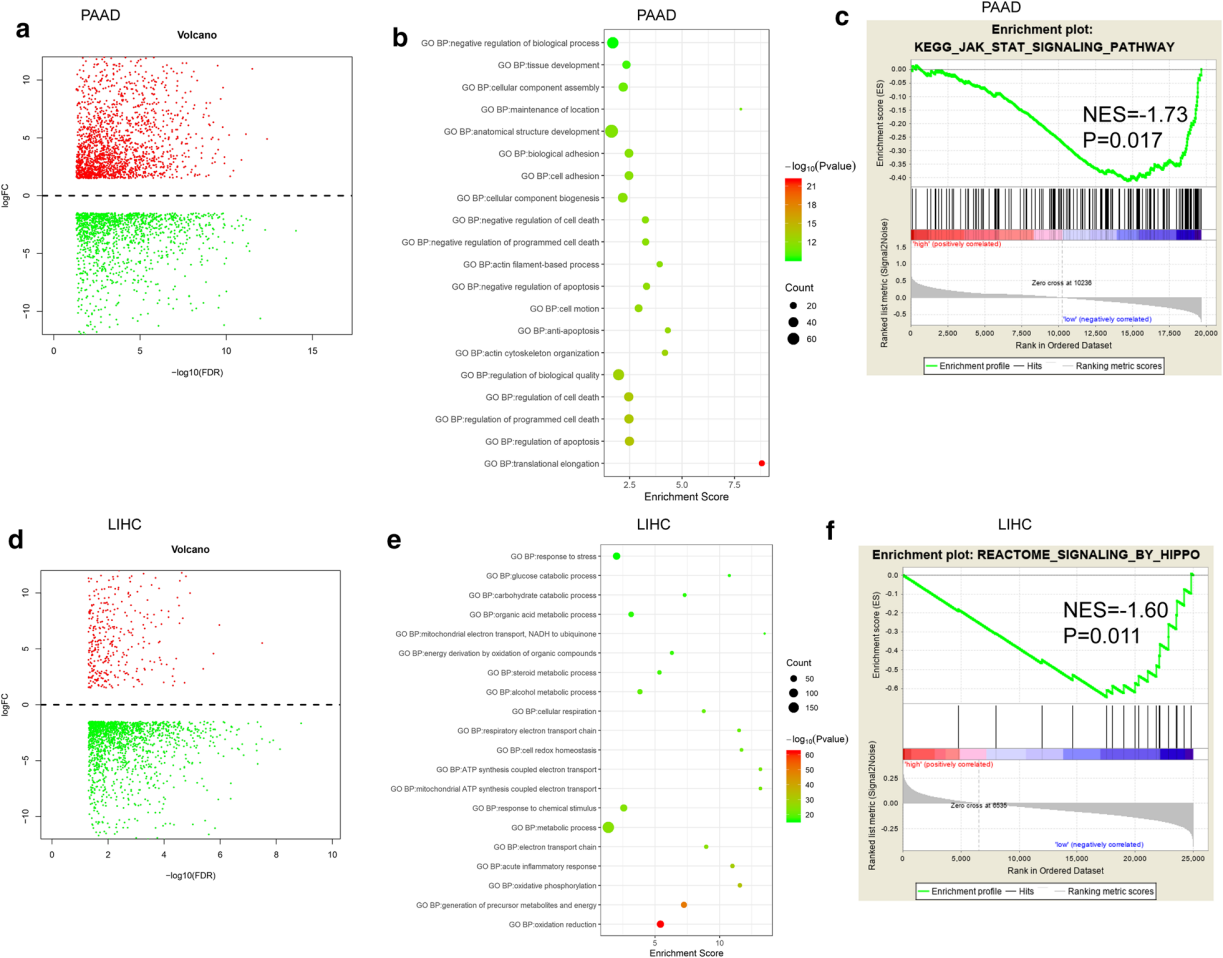
Differential gene and functional enrichment analysis of glycolysis metabolism–related signature. Volcano plots show the DEG in PAAD (**a**) and LIHC (**d**). GO functional annotations were performed based on the top 200 differential genes correlating positively with glycolysis metabolism in PAAD (**b**) and LIHC (**e**). GSEA analysis was based on the risk score in PAAD (**c**) and LIHC (**f**)

To search new possible links between glycolysis metabolism and biological function, we compared gene expression between the high- and low-risk groups based on the top 200 DEG (P < 0.05, ranked by fold change), displayed using volcano plots (Fig. 6a and d). The biological progression of the genes was annotated by GO, and we determined that negative regulation of cell death, biologic progression, inflammatory response, and metabolic process was enriched in the high-risk group (Fig. 6b and e). GO analysis was also used in GEO Validation set (Additional file 1:  Fig. S2a, b). GSEA showed that differential gene expression in the high- and low-risk groups was associated with the JAK–STAT pathway in PAAD, while the Hippo pathway was markedly related with LIHC (Fig. 6c, f). Based on glycolysis score, we analyzed all the differentially expressed genes, and then Screen out the STAT3 pathway related genes with differences in PAAD and Hippo pathway related genes with differences in LIHC. The top 10 genes were selected and shown in the histogram (Additional file 1: Fig. S3a, b). These biological functions may result in poor prognosis in the high-risk group.

### Targeting YAP1 in LIHC and STAT3 in PAAD can effectively inhibit glycolysis

**Fig. 7:**
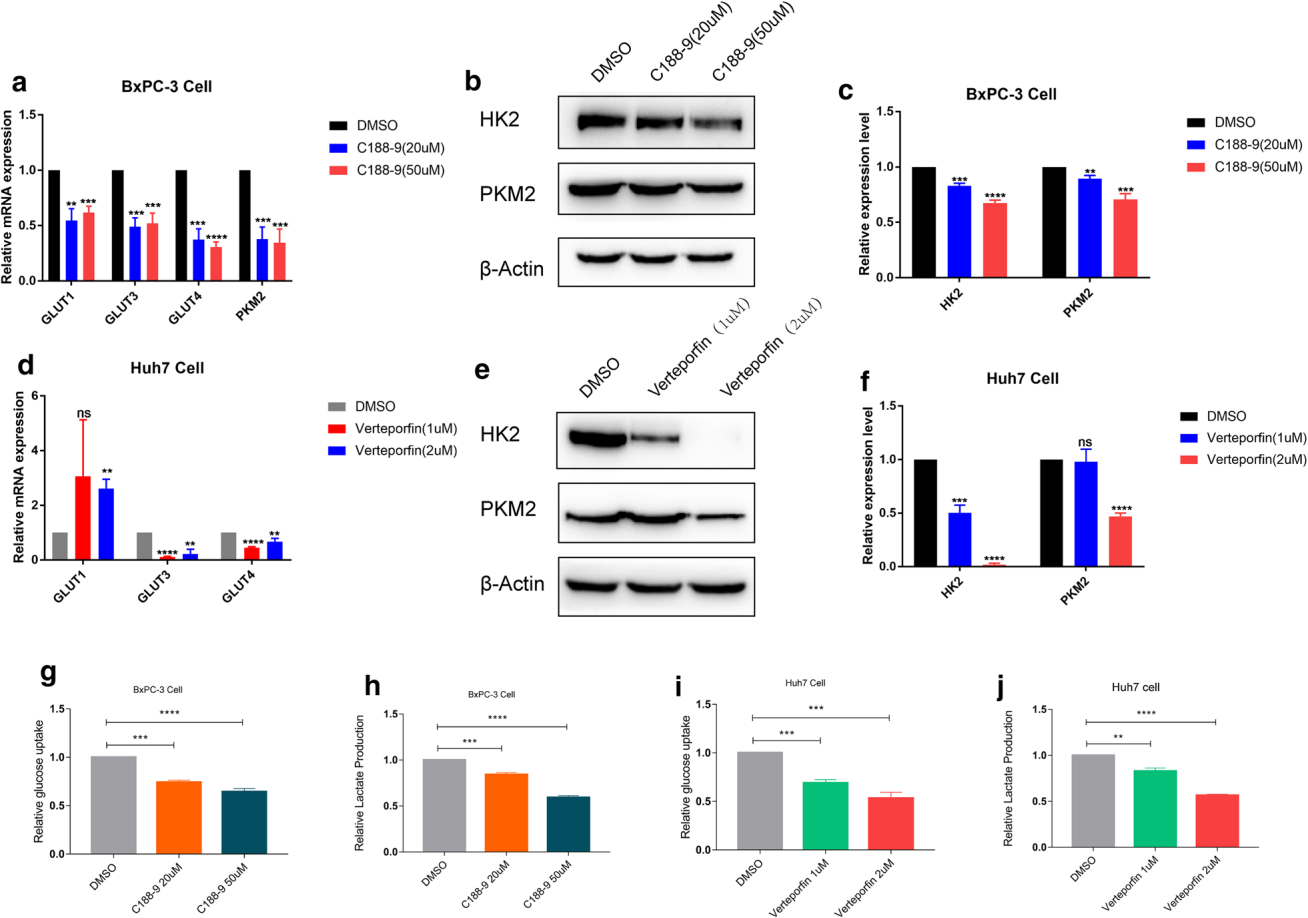
Inhibition of classical pathways can affect glycolysis metabolism. RT-PCR and Western Blotting were used to examine the glycolysis-related gene levels after STAT3 inhibitor (C188-9) treatment of BxPC-3 cells **(a-c)**, YAP1 inhibitor (verteporfin) treatment of Huh7 (**d–f**), respectively. Glycolysis uptake **(g, i)** and lactate formation **(h, j)** in the supernatant. **P < 0.01, ***P < 0.001, ****P < 0.0001, ns: no significance

We attempted to understand the relationship between glycolysis metabolism and the above signaling pathways. We downloaded TCGA RPPA protein database and stratified the PAAD patients into high P-STAT3 (high risk) group and low P-STAT3 (low risk) group based on the median value of P-STAT3, we also stratified the LIHC patients into high-YAP1 (high risk) and low-YAP1 (low risk) group. Then we compared the enrichment of glycolysis score as well as some classical glycolysis markers, we found an enhanced glycolysis status in high risk group, although some are not statistically significant (Additional file 1: Fig. S4a, b). We proposed that these pathways could regulate tumor aerobic glycolysis directly and verified this with experiments. First, we detected the inhibition efficiency of siYAP1 (Additional file 1: Fig. S6d-f) and verteporfin (Additional file 1: Fig. S6j-l) or C188-9 (Additional file 1: Fig. S6a-c) for pathways in LIHC and PAAD, respectively. We performed bioinformatic analyses in a few PAAD and LIHC cell lines by CCLE database. We found the glycolysis related genes of BxPC-3 in PAAD and Huh7 in LIHC have well expression level among these cell lines (Additional file 1: Fig. S5a, b). The STAT3 inhibitor (C188-9) significantly decreased the classical glycolysis-related mRNA levels in the BxPC-3 cells, such as *GLUT1* (solute carrier family 2 member 1), *GLUT3*, *GLUT4*, and *PKM2* (Fig. 7a), and also decreased HK2 and PKM2 in protein levels (Fig. 7b, c). Similar results were observed for LIHC with the YAP1 inhibitor verteporfin (Fig. 7d–f) or siYAP1 (Additional file 1: Fig. S6g-i). Accordingly, glucose uptake and lactate production also decreased after inhibitor treatment (Fig. 7g–j).

### In PAAD and LIHC, changes in glycolysis levels can affect the activity of Pathways

**Fig. 8:**
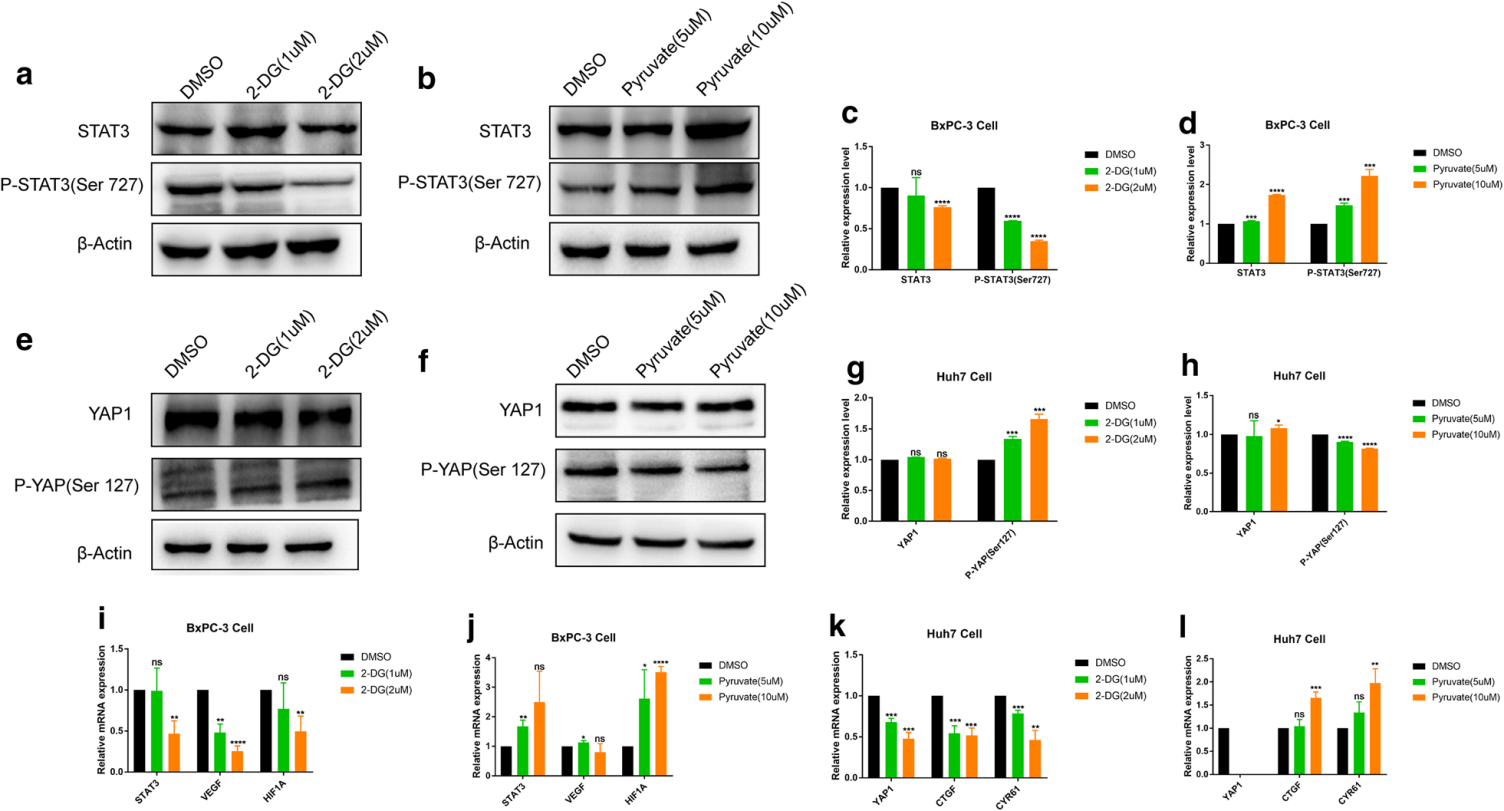
Glycolysis metabolism can affect the activation of classical pathways. BxPC-3 and Huh7 cells were treated with 2-DG or pyruvate, and western blotting detected STAT3 and YAP1 pathway activation, respectively (**a**–**h**), and RT-PCR detected Changes of downstream target genes (**i**–**l**). *P < 0.05, **P < 0.01, ***P < 0.001, ****P < 0.0001, *ns* no significance

To further investigate the effects of changes in glycolysis levels on the activity of YAP1 and STAT3 signaling pathways. The BxPC-3 and Huh7 cells were treated with 2-DG or pyruvate to inhibit or mimic the glycolysis effects, respectively. qPCR and Western Blotting were used to detect the inhibition or promotion efficiency of 2-DG or pyruvate on glycolysis both in PAAD (Additional file 1: Fig. S7a-f) and LIHC (Additional file 1: Fig. S7g-l). Interestingly, the level of glycolysis robustly influenced the activity of these two signaling pathways. We found that after the treatment of pancreatic cancer BxPC-3 cells with 2-DG in different concentrations, with the concentration increasing, the protein level of P-STAT3 decreased significantly (Fig. 8a, c), and qPCR showed that STAT3 and its downstream target genes, VEGF and HIF1A were also decreased (Fig. 8i). However, pyruvate significantly increased the expression of P-STAT3 (Fig. 8b and d) and the downstream target genes (Fig. 8j) in BxPC-3 cells. On the one hand, 2-DG significantly increased the expression of P-YAP in protein level (Fig. 8e and g) and decreased YAP1 and its downstream target genes, CTGF and CYR61 in mRNA level (Fig. 8k) in the liver cancer Huh7 cells. On the other hand, pyruvate could reduce the phosphorylation of YAP (Fig. 8f and h) and increase the expression of downstream target genes (Fig. 8l). These suggested that glycolysis level may in turn influence the activation of the pathway.

## Discussion

Increasing evidence suggests that metabolism status is closely related to the progression of malignant tumors. As a hallmark of cancer cells, glycolysis metabolism plays different roles in normal and cancer cells [5]. Normal cells generate energy through the tricarboxylic acid (TCA) cycle in aerobic conditions; under anaerobic conditions, glycolysis produces lactic acid. Under aerobic conditions, cancer cells undergo aerobic glycolysis, which may produce ATP at a rate of 100 times that of oxidative phosphorylation, thereby providing fast energy and providing more convenient conditions for tumor cells [21]. Here, we found that glycolysis metabolism status is closely related to the survival of patients with digestive system tumors. High glycolysis metabolism scores indicated poor prognosis. Further studies also showed that the glycolysis metabolism score in pancreatic adenocarcinoma cancer correlated with the patients’ clinicopathological characteristics. Increased PAAD grade was accompanied by increased glycolysis metabolism. In addition, high glycolysis metabolism scores also correlated with poor prognosis across the different PAAD grades.

Pathway regulation plays an important role in tumorigenesis and development [22, 23]. Here, glycolysis metabolism status was enriched in the JAK–STAT pathway in PAAD, and the same was true of the Hippo pathway in LIHC. However, the relationship between metabolic state and the pathways, whether metabolism affects pathway activation, whether the pathways regulate the metabolic state, or whether metabolism and pathways interact and regulate each other, ultimately leading to poor prognosis of digestive system cancer, remain unclear. In pancreatic cancer, JAK–STAT pathway inhibition can reduce the resistance and immune escape from chemotherapeutic drugs [24]. Pang et al. found that certain genes in the JAK–STAT pathway may be involved in poor prognosis of pancreatic cancer [25]. The upregulation of glycolysis can promote reactive oxygen species (ROS) production, targeting DCLK1 (doublecortin-like kinase 1) to promote the stem-like phenotype and epithelial–mesenchymal transition of gemcitabine-resistant pancreatic cancer cells [26]. Numerous studies have shown that the Hippo, WNT/β-catenin and Notch pathways play an important regulatory role in hepatocellular cancer development [27]. The activated Hippo pathway can inhibit monocyte/macrophage infiltration by inhibiting the expression of MCP1 (C-C motif chemokine ligand 2), limiting the infinite growth of hepatocytes [28]. Nevertheless, the concrete mechanism remains unclear. We found that the glycolysis metabolism status can affect the pathways, and that pathway inhibitors can reduce the glycolysis intake and lactic acid formation, suggesting that metabolism and the pathways may interact and regulate each other rather than a single aspect of regulation.

Cancer cells exhibit increased glycolysis, which is considered one of the most fundamental changes in tumor cell malignant transformation [29]. Currently, most antimetabolites are aimed at specific molecular targets of glycolysis, such as GLUT: blocking it can prevent glycolysis products from entering cancer cells, completely destroying the glycolysis pathways, and another common means of inhibiting glycolysis is targeting its key enzymes [30]. Due to patient heterogeneity and the pronounced instability in tumor occurrence and development, single chemotherapy drugs or anti-metabolic drugs do not have ideal therapeutic effects, or even generate tumor resistance [31]. Therefore, combining chemotherapy drugs and glycolytic inhibitors is a promising strategy for treating patients with cancer, and it has been extended to clinical treatment. Combined with our study, the use of combined pathway inhibitors may bring hope to patients with cancer.

## Conclusions

We conducted survival analysis of several digestive system tumors and found that glycolysis metabolism exhibited a highly stable survival prediction ability. Moreover, we found that the most critical signaling pathways in glycolysis modulation in PAAD and LIHC were STAT3 and YAP1, respectively. Interestingly, elevated glycolysis levels could enhance STAT3 and YAP1 activity in PAAD and LIHC cells, respectively, may existing a positive feedback loop. Our results may provide new insights into the indispensable role of glycolysis metabolism in digestive system tumors and guide the direction of future metabolism–signaling target combined therapy.

## Supplementary Information

**Additional file 1:** Supplementary Figures and Tables.

**Additional file 2:** Orginal data.

## Data Availability

All the databases we used are openly available in TCGA at http://cancergemome.nih.gov/, and GEO at http://www.ncbi.nlm.nih.gov/geo/.

## Abbreviations

PAAD: Pancreatic adenocarcinoma
LIHC: Liver Hepatocellular carcinoma
COAD: Colon adenocarcinoma
STAD: Stomach adenocarcinoma
GSVA: Gene set enrichment analysis
DEG: Differentially expressed genes
GO: Gene ontology
OS: overall survival
TCGA: The Cancer Genome Atlas
GEO: Gene Expression Omnibus
