# Supplementary material for: Identification of glycolysis related pathways in pancreatic adenocarcinoma and liver hepatocellular carcinoma based on TCGA and GEO datasets
Source: Cancer Cell Int. 2021 Feb 19;21:128. doi: 10.1186/s12935-021-01809-y (PMC7893943; doi:10.1186/s12935-021-01809-y)
Supplement: Supplementary file 1 — Additional file 1: Supplementary Figures and Tables. [file 12935_2021_1809_MOESM1_ESM.docx]

**Figure S1** The histogram showed the top 10 glycolytic genes which were the most significant differences in different glycolysis groups.

**
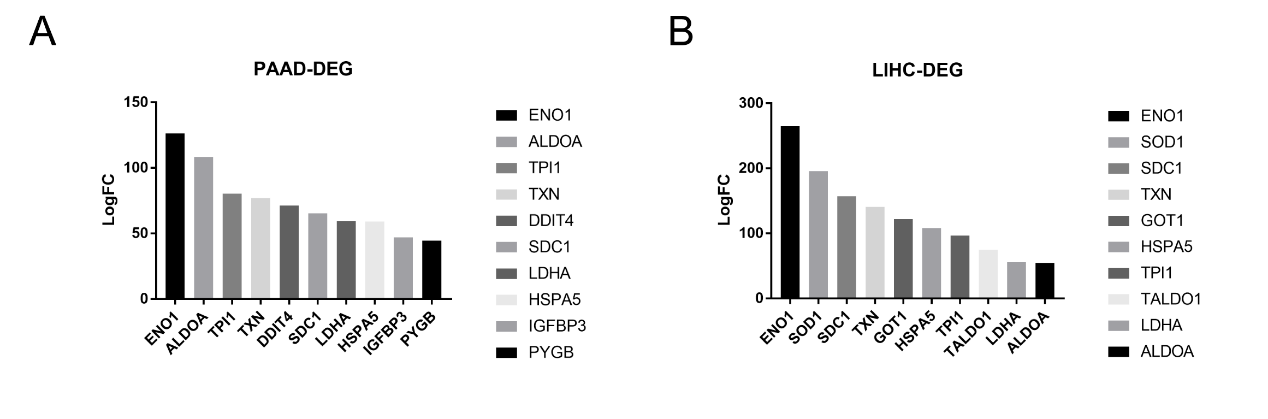
**

**Figure S2 Biological function analysis of GEO validation set. GO analysis was based on genes associated positively with glucose metabolism in PAAD (A) and LIHC (B).**


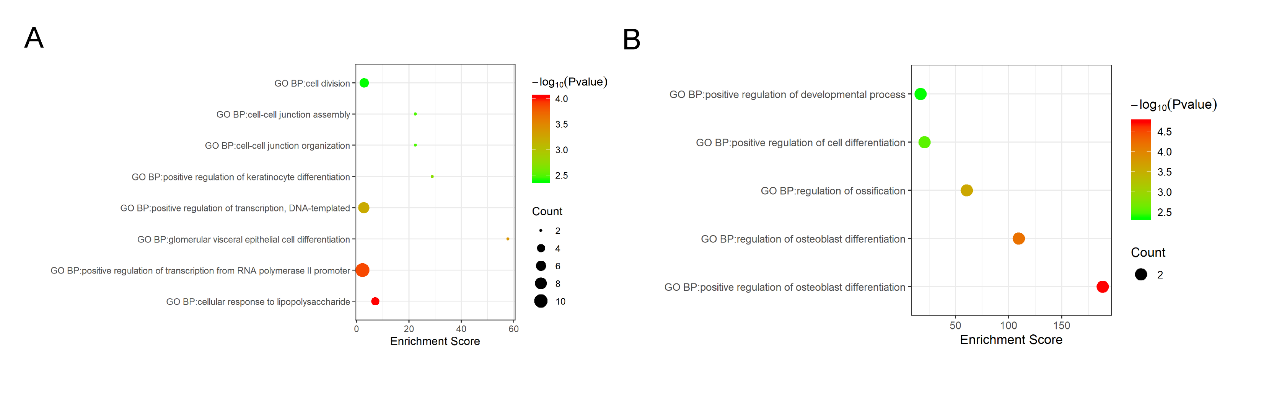


**Figure S3** The histogram showed the top 10 genes about STAT or Hippo pathway in PAAD (**A**) and LIHC (**B**), respectively, which were most differentially regulated by glycolysis.

**
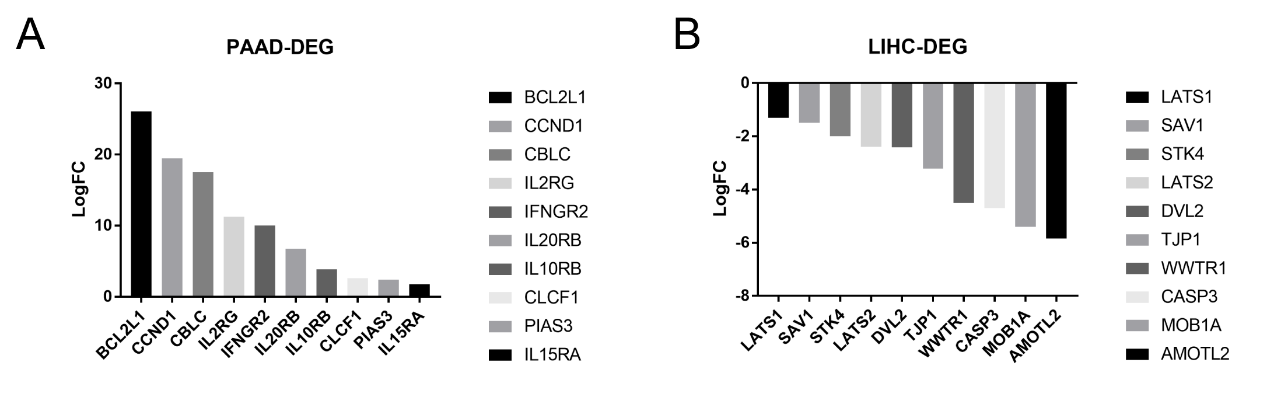
**

**Figure S4 Effects of STAT3 or YAP1 pathways on glycolysis related genes. According to the data in TCGA RPPA, PAAD (A) and LIHC (B) were grouped according to the median values of p-stat3 and YAP1, respectively, showing that the high P-STAT3 or YAP1 group also had high expression levels of glycolysis related genes.**

**
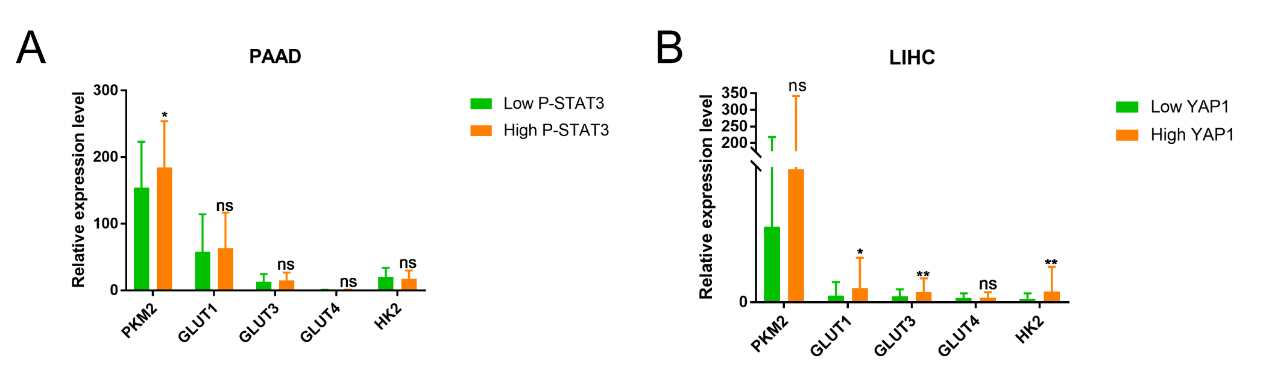
**

**Figure S5** The CCLE database was used to compare the expression of glycolysis related genes in some common PAAD (**A**) and LIHC (**B**) cell lines.

**
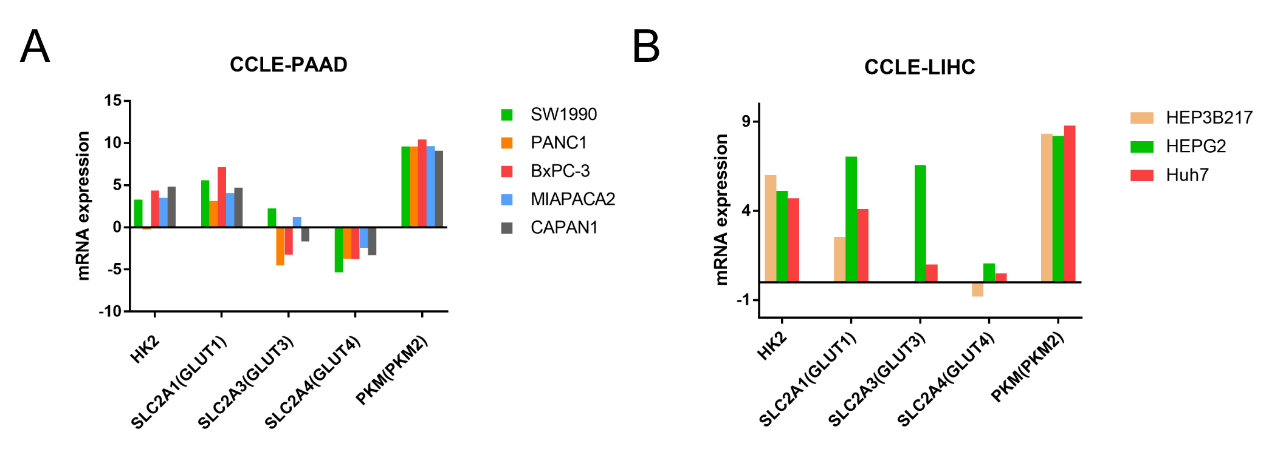
**

**Figure S6 Inhibition efficiency of siYAP1 and verteporfin or C188-9 for pathways in LIHC (A-F) and PAAD (G-I), respectively. siYAP1 can reduce the expression of glycolysis-related genes HK2 and PKM2 at the protein level (H-I) and reduce the GLUT1, GLUT3, GLUT4 and PKM2 expression at the mRNA level (G).**

**
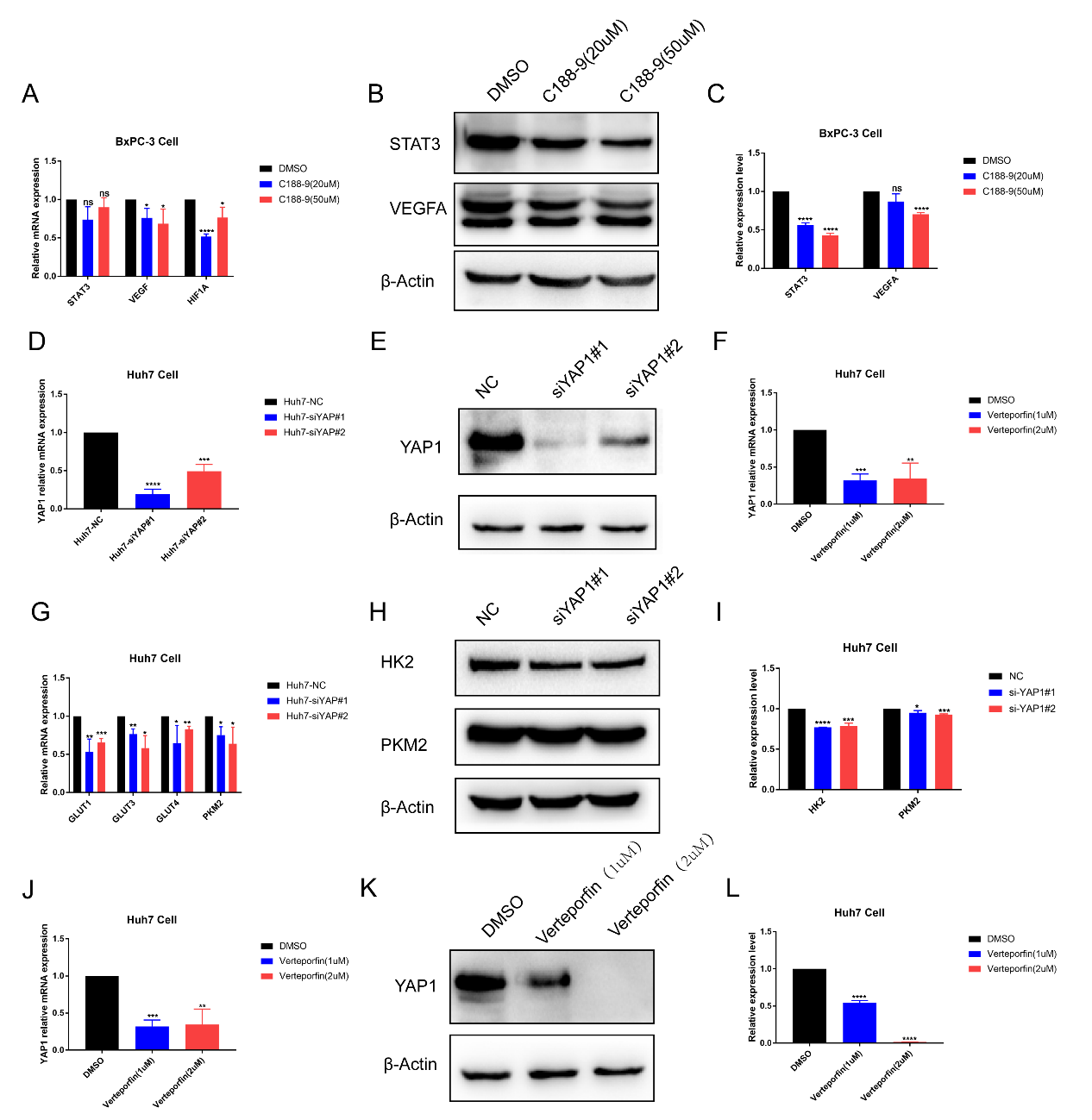
**

**Figure S7 Efficiency of 2-DG or pyruvate in glycolysis metabolism. RT-PCR and Western Blotting detected the glycolysis metabolism related markers after treatment with 2-DG or pyruvate in PAAD (A-F) and LIHC (G-L).**


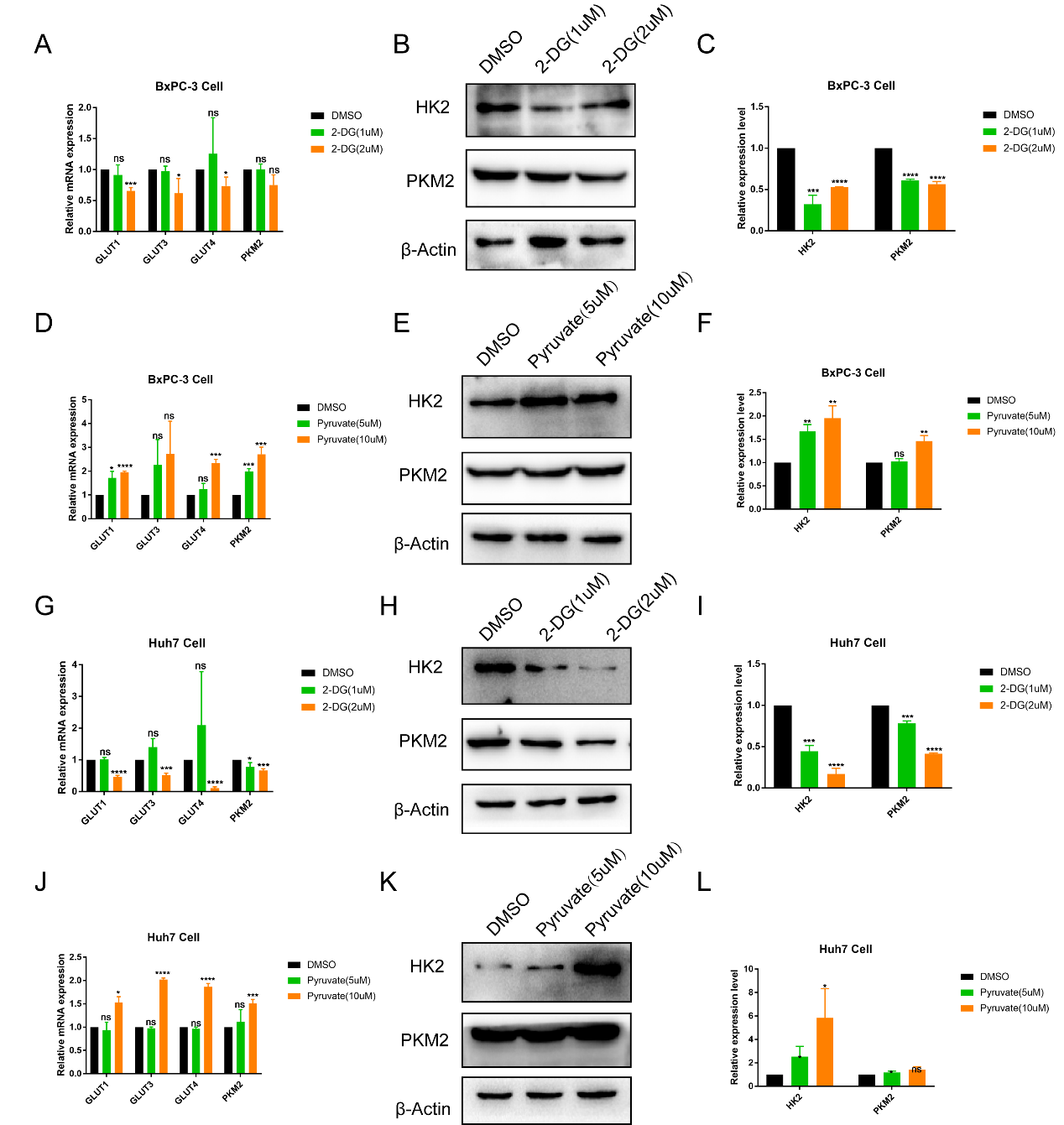


**Table S1.** Gene sets of the classical metabolism process.

| Glycolysis Metabolism | Fatty Acid Metabolism | Glutamine Metabolism |
| --- | --- | --- |
| ABCB6 | AADAT | AADAT |
| ADORA2B | ACAA1 | ADSSL1 |
| AGL | ACAA2 | AGMAT |
| AGRN | ACADL | ALDH18A1 |
| AK3 | ACADM | ALDH4A1 |
| AK4 | ACADS | ALDH5A1 |
| AKR1A1 | ACADVL | AMDHD1 |
| ALDH7A1 | ACAT2 | ARG1 |
| ALDH9A1 | ACO2 | ARG2 |
| ALDOA | ACOT2 | ART4 |
| ALDOB | ACOT8 | ASL |
| ALG1 | ACOX1 | ASNS |
| ANG | ACSL1 | ASNSD1 |
| ANGPTL4 | ACSL4 | ASRGL1 |
| ANKZF1 | ACSL5 | ASS1 |
| ARPP19 | ACSM3 | CAD |
| ARTN | ACSS1 | CPS1 |
| AURKA | ADH1C | CTPS |
| B3GALT6 | ADH7 | CTPS2 |
| B3GAT1 | ADIPOR2 | DAO |
| B3GAT3 | ADSL | DDAH1 |
| B3GNT3 | ALAD | DDAH2 |
| B4GALT1 | ALDH1A1 | FAH |
| B4GALT2 | ALDH3A1 | FPGS |
| B4GALT4 | ALDH3A2 | FTCD |
| B4GALT7 | ALDH9A1 | GAD1 |
| BIK | ALDOA | GAD2 |
| BPNT1 | AOC3 | GCLC |
| CACNA1H | APEX1 | GCLM |
| CAPN5 | AQP7 | GFPT1 |
| CASP6 | AUH | GFPT2 |
| CD44 | BCKDHB | GGH |
| CDK1 | BLVRA | GGT1 |
| CENPA | BMPR1B | GLS |
| CHPF | BPHL | GLS2 |
| CHPF2 | CA2 | GLUD1 |
| CHST1 | CA4 | GLUD2 |
| CHST12 | CA6 | GLUL |
| CHST2 | CBR1 | GMPS |
| CHST4 | CBR3 | GOT1 |
| CHST6 | CCDC58 | GOT2 |
| CITED2 | CD1D | HAL |
| CLDN3 | CD36 | LGSN |
| CLDN9 | CEL | MECP2 |
| CLN6 | CIDEA | MTHFS |
| COG2 | CPOX | NAGS |
| COL5A1 | CPT1A | NIT2 |
| COPB2 | CPT2 | NOS1 |
| CTH | CRAT | NOS2 |
| CXCR4 | CRYZ | NOS3 |
| CYB5A | CYP1A1 | NOXRED1 |
| DCN | CYP4A11 | OAT |
| DDIT4 | CYP4A22 | OTC |
| DEPDC1 | D2HGDH | PFAS |
| DLD | DECR1 | PHGDH |
| DPYSL4 | DHCR24 | PPAT |
| DSC2 | DLD | PRODH |
| ECD | DLST | PRODH2 |
| EFNA3 | ECH1 | PYCR1 |
| EGFR | ECHS1 | PYCR2 |
| EGLN3 | ECI1 | PYCRL |
| ELF3 | ECI2 | SIRT4 |
| ENO1 | EHHADH | SLC1A3 |
| ENO2 | ELOVL5 | TAT |
| ERO1L | ENO2 | UROC1 |
| EXT1 | ENO3 |  |
| EXT2 | EPHX1 |  |
| FAM162A | ERP29 |  |
| FBP2 | ETFDH |  |
| FKBP4 | FABP1 |  |
| FUT8 | FABP2 |  |
| G6PD | FASN |  |
| GAL3ST1 | FH |  |
| GALE | FMO1 |  |
| GALK1 | G0S2 |  |
| GALK2 | GABARAPL1 |  |
| GAPDHS | GAD2 |  |
| GCLC | GAPDHS |  |
| GFPT1 | GCDH |  |
| GLCE | GLUL |  |
| GLRX | GPD1 |  |
| GMPPA | GPD2 |  |
| GMPPB | GRHPR |  |
| GNE | GSTZ1 |  |
| GNPDA1 | H2AFZ |  |
| GOT1 | HADH |  |
| GOT2 | HADHB |  |
| GPC1 | HAO2 |  |
| GPC3 | HCCS |  |
| GPC4 | HIBCH |  |
| GPR87 | HMGCL |  |
| GUSB | HMGCS1 |  |
| GYS1 | HMGCS2 |  |
| GYS2 | HPGD |  |
| HAX1 | HSD17B10 |  |
| HDLBP | HSD17B11 |  |
| HK2 | HSD17B4 |  |
| HMMR | HSD17B7 |  |
| HOMER1 | HSDL2 |  |
| HS2ST1 | HSP90AA1 |  |
| HS6ST2 | HSPH1 |  |
| HSPA5 | IDH1 |  |
| IDH1 | IDH3B |  |
| IDUA | IDH3G |  |
| IER3 | IDI1 |  |
| IGFBP3 | IL4I1 |  |
| IL13RA1 | INMT |  |
| IRS2 | LDHA |  |
| ISG20 | LGALS1 |  |
| KDELR3 | LTC4S |  |
| KIF20A | MAOA |  |
| KIF2A | MCEE |  |
| LCT | MDH1 |  |
| LDHA | MDH2 |  |
| LDHC | ME1 |  |
| LHPP | METAP1 |  |
| LHX9 | MGLL |  |
| MDH1 | MIF |  |
| MDH2 | MLYCD |  |
| ME1 | NBN |  |
| ME2 | NCAPH2 |  |
| MED24 | NSDHL |  |
| MERTK | NTHL1 |  |
| MET | ODC1 |  |
| MIF | OSTC |  |
| MIOX | PCBD1 |  |
| MPI | PDHA1 |  |
| MXI1 | PDHB |  |
| NANP | PPARA |  |
| NASP | PRDX6 |  |
| NDST3 | PSME1 |  |
| NDUFV3 | PTPRG |  |
| NOL3 | PTS |  |
| NSDHL | RAP1GDS1 |  |
| NT5E | RDH11 |  |
| P4HA1 | RDH16 |  |
| P4HA2 | REEP6 |  |
| PAM | RETSAT |  |
| PAXIP1 | S100A10 |  |
| PC | SDHA |  |
| PDK3 | SDHC |  |
| PFKFB1 | SDHD |  |
| PFKP | SERINC1 |  |
| PGAM1 | SETD8 |  |
| PGAM2 | SLC22A5 |  |
| PGK1 | SMS |  |
| PGLS | SUCLA2 |  |
| PGM2 | SUCLG1 |  |
| PHKA2 | SUCLG2 |  |
| PKM2 | TDO2 |  |
| PKP2 | TP53INP2 |  |
| PLOD1 | UBE2L6 |  |
| PLOD2 | UGDH |  |
| PMM2 | UROD |  |
| POLR3K | UROS |  |
| PPFIA4 | VNN1 |  |
| PPIA | XIST |  |
| PPP2CB | YWHAH |  |
| PRPS1 |  |  |
| PSMC4 |  |  |
| PYGB |  |  |
| PYGL |  |  |
| QSOX1 |  |  |
| RARS |  |  |
| RBCK1 |  |  |
| RPE |  |  |
| RRAGD |  |  |
| SAP30 |  |  |
| SDC1 |  |  |
| SDC2 |  |  |
| SDC3 |  |  |
| SDHC |  |  |
| SLC16A3 |  |  |
| SLC25A10 |  |  |
| SLC25A13 |  |  |
| SLC35A3 |  |  |
| SLC37A4 |  |  |
| SOD1 |  |  |
| SOX9 |  |  |
| SPAG4 |  |  |
| SRD5A3 |  |  |
| STC1 |  |  |
| STC2 |  |  |
| STMN1 |  |  |
| TALDO1 |  |  |
| TFF3 |  |  |
| TGFA |  |  |
| TGFBI |  |  |
| TKTL1 |  |  |
| TPBG |  |  |
| TPI1 |  |  |
| TPST1 |  |  |
| TSTA3 |  |  |
| TXN |  |  |
| UGP2 |  |  |
| VCAN |  |  |
| VEGFA |  |  |
| VLDLR |  |  |
| XYLT2 |  |  |
| ZNF292 |  |  |

**Table S2.** Sequences of siRNAs

| siRNA Names | Sequences |
| --- | --- |
| siNC | UUCUCCGAACGUGUCACGUTT |
|  | ACGUGACACGUUCGGAGAATT |
| siYAP#1 | GCUCAUUCCUCUCCAGCUUdTdT |
|  | AAGCUGGAGAGGAAUGAGCdTdT |
| siYAP#2 | CACCUAUCACUCUCGAGAUdTdT |
|  | AUCUCGAGAGUGAUAGGUGdTdT |
