# Supplementary figures and images for: Identification of glycolysis related pathways in pancreatic adenocarcinoma and liver hepatocellular carcinoma based on TCGA and GEO datasets
Source: Cancer Cell Int. 2021 Feb 19;21:128. doi: 10.1186/s12935-021-01809-y (PMC7893943; doi:10.1186/s12935-021-01809-y)

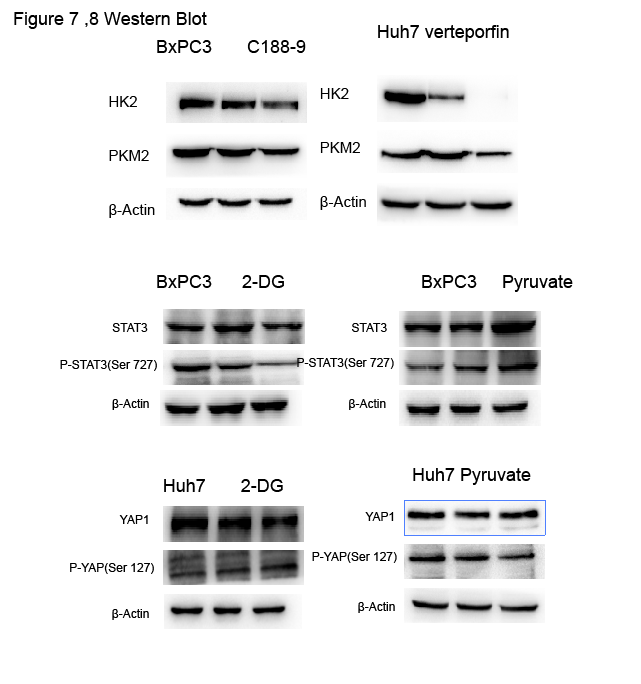


PCR

BxPC3


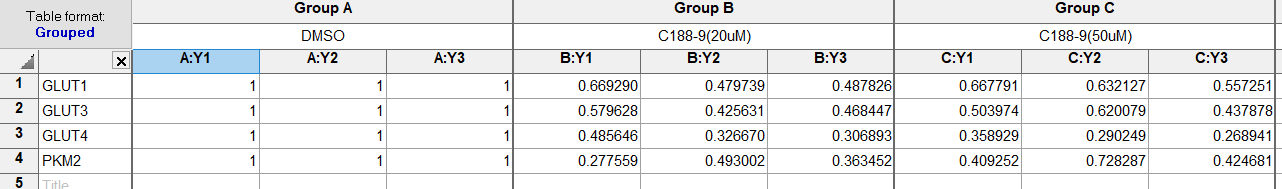


Huh7


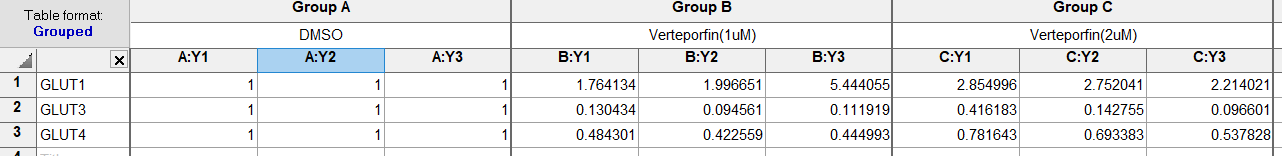


BxPC3


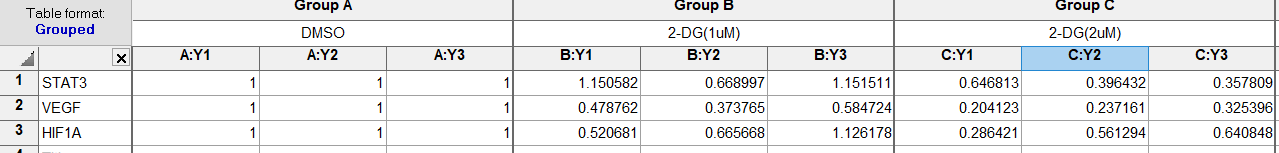

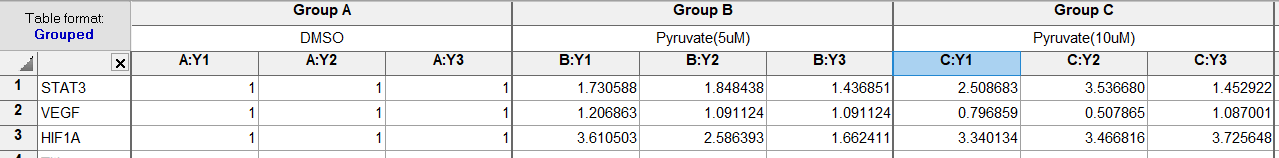


Huh7


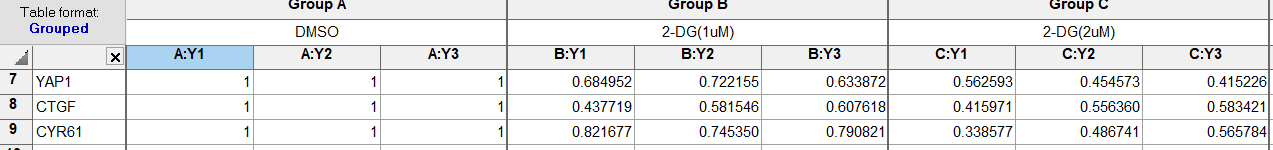


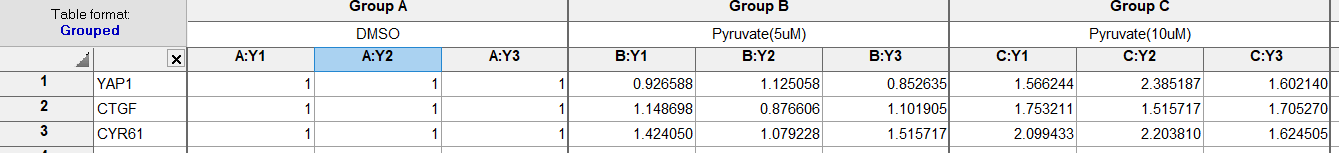


BxPC3


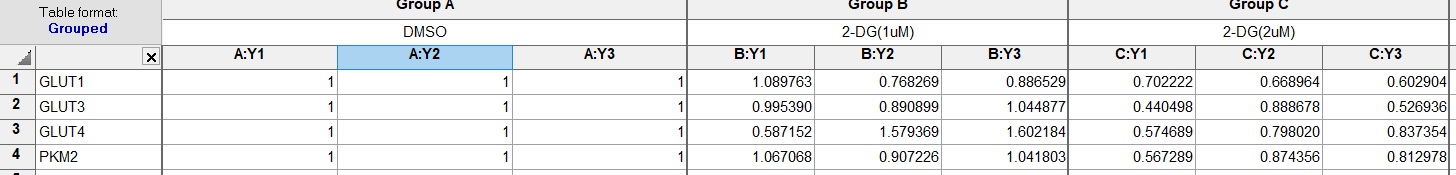


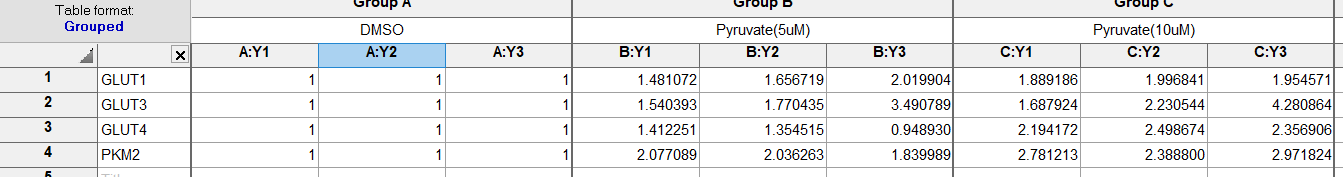


Huh7


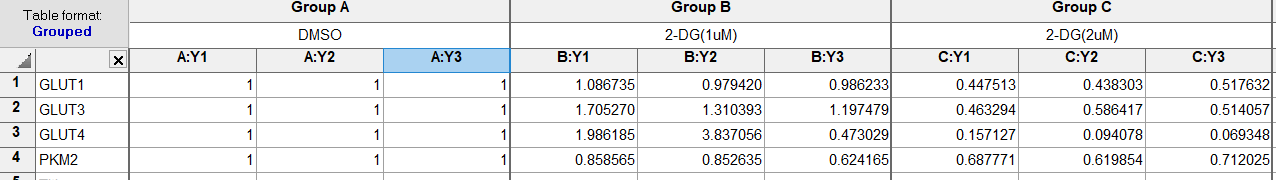


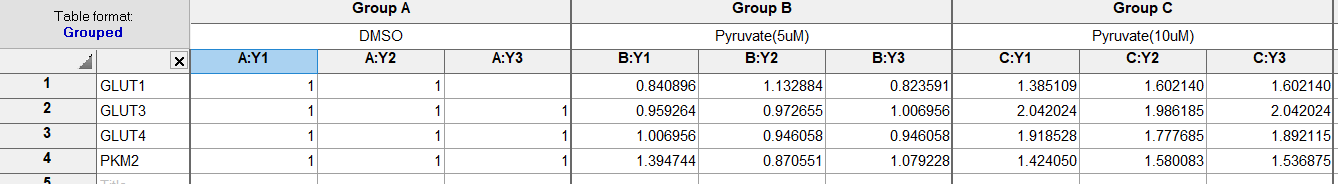

Supplement: Supplementary file 2 — Additional file 2: Orginal data. [file 12935_2021_1809_MOESM2_ESM.docx]
